# PatchEpi: patch-aware equivariant learning improves structure-based epitope prediction

**DOI:** 10.1093/bioinformatics/btag678

**Published:** 2026-09-15

**Authors:** Sicheng Wen, Fei Li, Yue Qian

**Affiliations:** MARS Department, Viva Biotech (Shanghai) Limited, Shanghai 201318, China; MARS Department, Viva Biotech (Shanghai) Limited, Shanghai 201318, China; MARS Department, Viva Biotech (Shanghai) Limited, Shanghai 201318, China

## Abstract

**Motivation:**

Accurate prediction of B-cell epitopes is essential for antibody design and vaccine development, yet remains fundamentally challenging. A major but often overlooked limitation of existing predictors is their implicit assumption that epitope identity can be decomposed into independent residue-level signals. In contrast, antibody recognition is governed by spatially contiguous surface patches whose functional identity emerges from coherent three-dimensional geometry rather than isolated residues.

**Results:**

Here, we reformulate structure-based B-cell epitope prediction as a surface-patch learning problem and introduce PatchEpi, a patch-aware geometric deep learning framework for antigen-intrinsic epitope modeling. It employs a patch-aware attention encoder and boundary-contrastive objectives to align the learning patch signal with the biological reality of antibody binding. PatchEpi integrates patch embeddings, fine-tuned ESM2 representations, and an equivariant message-passing network to model the 3D topology of antigen surfaces. To enable rigorous evaluation, we created a homology leakage-controlled split by reclustering the ANABAG dataset. Across multiple benchmarks, PatchEpi consistently outperforms residue-centric and graph-based state-of-the-art methods. Structural analyses further show that the model learns coherent surface patches that closely match experimentally resolved antibody footprints. These results demonstrate that accurate epitope prediction benefits primarily from reformulating the task around surface-patch learning. Patch-aware learning provides a principled and biologically grounded pathway toward more reliable structure-based epitope detection.

**Availability and implementation:**

The code and related resources are available at GitHub (https://github.com/wsicheng739/PatchEpi) and Zenodo (https://doi.org/10.5281/zenodo.20366444).

## 1 Introduction

Accurate identification of conformational epitopes is essential for antibody design, vaccine engineering, and therapeutic development. Critically, epitope recognition is inherently a patch-level process (Parvizpour *et al.* 2020, De Leon *et al*. 2024). Antibodies do not bind isolated residues; instead, they engage contiguous, spatially coherent surface regions whose identity emerges from solvent exposure, local curvature, and the geometric arrangement of neighboring residues (Kringelum *et al.* 2013, Peng *et al.* 2014, Lu *et al.* 2022, Tubiana *et al*. 2022, Zeng X *et al.* 2023). Despite this well-established biological view, most computational methods continue to frame epitope prediction as an independent residue-level classification task (Zhou *et al.* 2019, Silva *et al.* 2022, Choi and Kim 2024, Høie *et al.* 2024, Wang *et al.* 2025). This formulation overlooks the fact that residues within the same epitope share consistent structural and physicochemical context, whereas residues across the epitope boundaries exhibit abrupt geometric transitions. As a result, existing models frequently produce scattered false positives and fragmented predictions that poorly resemble true antibody binding interfaces.

Recent advances in protein language models such as ESM2 (Lin *et al.* 2023) have substantially improved residue-level representations. However, these models do not inherently capture the geometric continuity that characterizes epitope surfaces. Sequence-based methods like BepiPred-3.0 (Clifford *et al.* 2022) often highlight exposed or flexible residues, yet they tend to disperse these signals across the antigen surface. Similarly, geometric learning approaches such as GraphBepi (Zeng Y *et al.* 2023) exhibit inherent limitations in reliably determining whether identified epitope-like residues belong to the same structural patch. Consequently, their outputs are spatially inconsistent or fragmented, failing to reflect the contiguous nature of true antibody-binding interfaces.

Crucially, the issue of data leakage, specifically the evaluation bias arising from residual homology between training and testing sets, remains under-addressed in the epitope prediction literature. Although critical reviews have highlighted this issue (Cia *et al*. 2023, Yu *et al*. 2025), state-of-the-art epitope predictors report only the sequence identity cutoff applied during MMseqs2 clustering, without further analysis of residual redundancy. The availability of a large number of antigen-antibody complexes in ANABAG (Grandguillaume *et al*. 2025) provides an opportunity to systematically investigate the impact of data leakage on epitope prediction performance.

To address these limitations, we propose a patch-aware epitope prediction framework that models the spatial topology of antibody-antigen interfaces. In this formulation, a patch is defined as a surface region in which spatially connected residues exhibit coherent predictive behavior. Our architecture integrates ESM2-derived embeddings with an Equivariant Graph Neural Network (EGNN) (Garcia Satorras *et al*. 2021) and a Patch-Coherence encoder that learns latent representations of local antigen neighborhoods. The resulting patch embeddings generate a patch-similarity signal that modulates EGNN message-passing weights, thereby reinforcing smooth and coherent predictions within epitope regions while preserving sharp boundaries at epitope-non-epitope interfaces. By treating epitopes as integrated three-dimensional surface patches, this approach aligns the learning objective closer to the biological mechanism of antibody recognition. Evaluated on a rigorously curated, leakage-controlled split of ANABAG and the Epitope3D (Silva *et al.* 2022) blind test set, our model achieves consistent improvements in Matthews correlation coefficient (MCC), Area under the precision-recall curve (AUPRC) and Area under the ROC curve (ROC-AUC) over existing approaches.

By reframing epitope prediction in terms of surface patches, this study introduces a biologically grounded and geometrically principled framework that more faithfully reflects how antibodies recognize antigens. Our findings highlight that the spatial organization of residues is essential for accurate computational epitope prediction.

## 2 Methods

### 2.1 ANABAG dataset

Selecting a diverse and biologically meaningful dataset is crucial for developing high-quality B-cell epitope prediction models. Traditional predictors such as BepiPred-3.0 are typically trained on small and curated benchmark datasets. In contrast, our model leverages ANABAG, containing 6948 antibody-antigen complexes that span diverse antigen classes, including viruses (e.g. SARS-CoV-2, HIV), GPCRs, and many others. ANABAG provides residue-level annotations derived from solvent-accessible surface area (SASA) differences between bound and unbound states, along with machine-learning-ready structural features. To rigorously assess model generalization, we further employ the Epitope3D blind test set, which consists of 45 unbound antigen structures and is widely recognized within the epitope prediction community as a standard external benchmark.

### 2.2 Dataset pre-processing

Given the complexity of ANABAG, we implemented a rigorous data-cleaning pipeline to exclude entries with incomplete antigen structures, chain mismatches, residue numbering inconsistencies, or missing coordinates. Epitope residues were defined using a standard SASA-based criterion (ΔSASA > 1.0 Å^2^), consistent with the annotation protocol adopted in ANABAG, and this definition was used as the ground truth for the ANABAG internal test set.

Because ANABAG does not provide predefined data splits, we constructed our own train, validation and internal-test split. To prevent data leakage and ensure biological separation between antigen families across the ANABAG training set, the internal ANABAG test set, and the external Epitope3D test set, we performed joint clustering of 6724 ANABAG antigens and 45 Epitope3D antigens using MMseqs2 (Steinegger and Söding 2017) at 60% sequence identity threshold. Entire clusters were assigned to a single data split, thereby preventing highly similar or homologous antigens from being distributed across training, validation, and test partitions. To preserve a consistent 9:1 train-test ratio, several small clusters were relocated into the test split. The 45 Epitope3D antigens served as the external benchmark, and any ANABAG antigens clustering with these test structures were excluded from training to guarantee strict separation. Leakage was assessed using sequence identity, not a generic sequence similarity score, together with alignment coverage criteria: sequence identity ≥ 60%, query coverage ≥ 80%, and target coverage ≥ 80%. This strategy produced leakage-controlled training, internal testing, and external evaluation sets while preserving comparability with existing structure-based epitope prediction methods. The consequences of alternative splitting strategies (e.g. random or non-clustered splits) were emphasized in the Results section. Further information on the datasets employed in this study is available in Supplementary Note 1 and 2.

### 2.3 Model architecture

**Figure 1:**
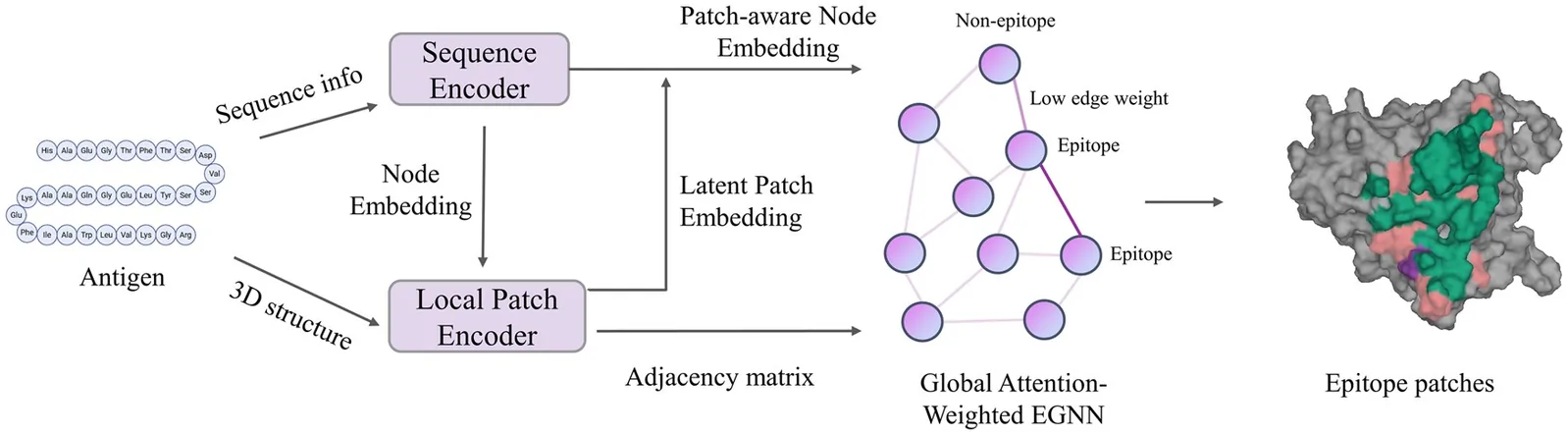
Overview of the proposed patch-aware epitope prediction framework. The antigen sequence was processed by a sequence encoder (ESM2-150M with fine-tuning) to capture global contextual information, while a local Patch-Coherence encoder (a graph encoder operating on 8 Å neighborhoods) learned latent representations of structurally coherent patches. These sequence- and patch-level embeddings were integrated within a geometric graph and propagated through an EGNN, where patch-similarity scores dynamically modulate edge attention. This patch-aware message passing encouraged prediction consistency within epitope regions while maintaining sharp boundaries across patch edges. The final residue-level outputs were assembled into contiguous surface patches.

Epitopes typically manifest as local, spatially coherent 3D surface patches, and our framework, as shown in Fig. 1, is designed to mirror these properties by enforcing sequence-informed epitope supervision, patch-level coherence constraints and radius-restricted message passing.

Each antigen chain was first processed by ESM2-150M encoder to generate context-aware residue embeddings. In contrast to existing structure-based predictors which operated on isolated chains, our method explicitly preserved the multi-chain organization of the antigen. Residue embeddings (h_i) from all chains were concatenated into a unified sequence, with chain-identity embeddings incorporated to indicate chain boundaries, which is expressed as the following equation. Detailed explanation of each symbol in the equation is shown in Table S3.

Local geometric information was encoded by a Patch-Coherence Graph Auto-Encoder (GAE) operating on strict 8 Å neighborhoods, a commonly used cutoff for defining spatial residue contacts in protein structures (Bhattacharya and Bhattacharya 2020, Xie and Xu 2022, Réau *et al.* 2023). Sensitivity analysis with alternative cutoffs supported the robustness of this setting (Table S4).


(1) $$(\mu_{i},\log\;\sigma_{i})=\mathrm{PatchEnc}{\left(x^{\left(0\right)},E_{\text{patch}}\right)}_{i}$$

(2) $$z_{i}=\mu_{i}+\sigma_{i}⊙\epsilon ,\epsilon ∼N(0,\;I)$$

These patch features were subsequently fused with the sequence embeddings and processed by an EGNN built on a residue-residue proximity graph. Message passing was further modulated by a patch-similarity edge-attention mechanism, which reinforced information flow within coherent surface patches while suppressing interactions across patch boundaries.


(3) $$s_{ij}=\langle {\tilde{z}}_{i},{\tilde{z}}_{j}\rangle$$

(4) $$w_{ij}=\sigma ({\hat{\alpha }}_{ij})$$

EGNN message:


(5) $$m_{ij}^{\left(\ell \right)}=w_{ij}\cdot \phi_{m}\left(x_{i}^{\left(\ell \right)},x_{j}^{\left(\ell \right)},{∥r_{i}-r_{j}∥}_{2}\right)$$

EGNN node update:


(6) $$x_{i}^{\left(\ell +1\right)}=x_{i}^{\left(\ell \right)}+\sum_{j\in N(i)}\;w_{ij}m_{ij}^{\left(\ell \right)}$$

The final prediction head produced residue-level binding probabilities. The loss function was designed to achieve the same goal as Patch-Coherence Encoder and gating mechanism. It consists of three components: weighted Binary Cross-Entropy (BCE) loss, Laplacian loss and contrastive boundary loss (Kervadec *et al.* 2019, Li *et al*. 2020). Further elaborations are detailed in Supplementary Note 5.


(7) $$L_{\mathrm{BCE}}=-\sum_{i}\;(w_{1}y_{i}l\mathrm{og}{\hat{y}}_{i}+w_{0}(1-y_{i})l\mathrm{og}(1-{\hat{y}}_{i}))$$

(8) $$L_{\text{smooth}}=\frac{1}{\left|E\right|}\sum_{(i,j)\in E}\;{\left({\hat{y}}_{i}-{\hat{y}}_{j}\right)}^{2}$$

(9) $$L_{\mathrm{Ct}r\;\text{boundary}}=\frac{1}{\left|B\right|}\sum_{(i,j)\in B}\;max(0,m-(p_{ij}^{+}-p_{ij}^{-}))\;\text{with}\;m=0.3$$

(10) $$L=\lambda_{\mathrm{BCE}}L_{\mathrm{BCE}}+\lambda_{\text{smooth}\;}L_{\text{smooth}}+\lambda_{\text{boundary}\;}L_{\text{boundary}\;}$$

### 2.4 Node and edge embeddings

Each residue in the antigen graph carried complementary node features that captured sequence context, local geometry, chain identity, and patch-level structure. Specifically, a 128-dimensional sequence embedding was derived for each residue. To incorporate surface topology, two geometric descriptors, normalized neighbor count and local curvature, were computed from the 3D coordinates. A 16-dimensional chain embedding encoded chain identity, allowing residues from different chains to be distinguished. Finally, a 64-dimensional latent vector from the patch encoder captured each residue’s membership within local structural patches. These features were concatenated into a unified hidden representation that served as the input for subsequent EGNN propagation.

Edges in the graph encoded both geometric connectivity and a learned attention mechanism that guided how information flows across the antigen surface. We constructed the graph by linking residue pairs within an 8 Å radius to capture local structural contacts. On each edge, the model computed a patch-similarity attention weight derived from the cosine similarity of latent patch embeddings. This attention gate selectively amplified message passing between residues that shared a coherent structural patch while constraining communication across unrelated or discontinuous regions. In parallel, the EGNN incorporated squared coordinate distances as an equivariant geometric term, ensuring that attention was grounded in physical spatial relationships.

### 2.5 Model training and evaluation

To maximize the effective training size, the original training and validation subsets were combined into a single training pool. For each training run, this pool was partitioned into 90% training and 10% validation data. Crucially, the independent test set remained strictly untouched and was used exclusively for final performance evaluation.

During training, we optimized a class-balanced binary cross-entropy loss, where the positive class weight was derived from the global antigen-level epitope ratio. In addition, boundary loss and smooth loss regularizers were formulated over pairs of neighboring residues, penalizing large discrepancies in predicted epitope probabilities among residues sharing the same label while encouraging contrast between epitope and non-epitope pairs. Optimization was performed using the AdamW optimizer (learning rate = 0.001, batch size = 6). Training was conducted for 15 epochs, and the model checkpoint achieving the highest validation MCC was selected for final evaluation.

The Epitope3D benchmark set and the internal ANABAG test set were used for the final assessment of generalization performance. These test sets were strictly excluded from all stages of training, validation, and threshold selection. All reported performance metrics, including MCC (primary metric), ROC-AUC, AUPRC, precision, recall and balanced accuracy (BACC) were calculated exclusively on these held-out test sets.

To compare PatchEpi with other methods on the Epitope3D blind test set, we submitted the corresponding antigen structures to the available web servers. Unless otherwise specified, the reported metrics were calculated using each server’s default prediction threshold. We selected EpiGraph as the retraining baseline because it was originally trained and evaluated in the Epitope3D setting (Choi and Kim 2024). Since the Epitope3D blind test is used as the benchmark testing dataset in this study, retraining EpiGraph on ANABAG enabled us to assess the effect of training-set choice under a consistent evaluation benchmark. During re-training, the original EpiGraph architecture and all default hyper-parameters were kept unchanged; the only modification involved adapting the ANABAG dataset into the input format required by EpiGraph. Both models were evaluated against Epitope3D blind test set.

## 3 Results

### 3.1 Model performance on Epitope3D test set

Table 1 details the comparative performance of PatchEpi against several representative epitope prediction methods on the Epitope3D blind test set (45 antigens). To avoid inconsistencies introduced by retraining external models on non-identical datasets, we evaluated baseline methods using their publicly available web servers, without retraining. Unless otherwise specified, binary predictions were generated using each method’s default threshold, and all metrics were recalculated using the same Epitope3D residue-level annotations and script. For consistency across methods, Table 1 reports all-residue evaluation for all models without applying RSA-based (relative solvent accessible area) filters. As a sensitivity analysis, we additionally re-evaluated the Epitope3D predictions after applying an RSA ≥ 15% surface-residue filter.

**Table 1:** Comparison of PatchEpi and existing epitope prediction methods on the Epitope3D blind test set comprising 45 antigens.

Model	Threshold	MCC	AUPRC	AUC	Precision	Recall	BACC
Epitope 3D	0.500^a^	−0.01	0.04	0.49	0.03	0.13	0.49
SEPPA 3.0	0.089	0.06	0.09	0.62	0.10	0.42	0.55
SEMA 2.0	0.510	0.14	0.17	0.70	0.11	**0.78**	0.63
Discotope-3.0	0.900	0.14	0.12	0.68	0.14	0.39	0.61
BepiPred-3.0	0.151	0.18	0.18	0.75	0.14	0.59	0.66
EpiGraph	–	0.20	0.20	0.76	0.21	0.36	0.63
GraphBepi	0.176	0.20	0.23	0.74	0.18	0.53	0.65
RoBep	0.353	0.23	0.23	0.67	**0.22**	0.44	0.66
PatchEpi	0.495	**0.26**	**0.25**	**0.79**	0.21	0.58	**0.70**

External baseline methods were evaluated using their publicly available web servers with default parameters, without retraining. All metrics were recalculated using the same Epitope3D residue-level annotations and script. Bold values indicate the best performance for each evaluation metric across all compared methods.

a Epitope 3D provided only binary epitope predictions without residue-level continuous scores. Thus, predicted epitope residues were encoded as 1 and all others as 0; the 0.5 threshold was used only to define these binary labels.

Because residue-level epitope prediction is highly class-imbalanced, MCC was used as the primary evaluation metric. Based on MCC, Epitope3D and SEPPA 3.0 showed weak performance, SEMA 2.0 (Ivanisenko *et al.* 2024), DiscoTope-3.0, and BepiPred-3.0 showed moderate performance, whereas EpiGraph, GraphBepi (Zeng Y *et al.* 2023), RoBep (Xu *et al.* 2026), and PatchEpi showed relatively stronger performance. Additionally, Epitope3D consistently exhibited weak performance after using RSA ≥ 15% surface-residue filter (MCC = 0.02, AUC = 0.51, AUPRC = 0.04, precision = 0.14, recall = 0.04). This overall trend is consistent with the benchmark study by Cia, Pucci, and Rooman, who reported generally limited performance of existing conformational B-cell epitope prediction webservers, with some methods performing close to random surface-patch baselines (Cia *et al*. 2023). PatchEpi achieved the highest MCC of 0.26, outperforming RoBep (0.23), GraphBepi (0.20), and EpiGraph (0.20), while also maintaining competitive performance across AUPRC, AUC, and balanced accuracy. Additional evaluation on the RoBep 144-antigen test set is provided in Table S5; MMseqs2 analysis suggested that this test set was substantially covered by sequences represented in the PatchEpi training set. Therefore, we focused on fully independent benchmarks (Epitope3D blind test) for the main evaluation. Following the recent RoBep study, which has modelled epitopes as spatially coherent surface patches, we also calculated clustered mean pairwise distance (cMPD) to assess the spatial compactness of predicted epitopes. On the Epitope3D blind test set, PatchEpi achieved a lower cMPD than RoBep (16.98 Å versus 18.09 Å), demonstrating more compact spatial predictions.

To ensure a fair and conservative evaluation, we recognized the necessity of retraining top-performing models, as they were originally developed and optimized using different datasets. EpiGraph therefore was selected and retrained with the default architecture and hyperparameters; the only change was converting ANABAG into the required EpiGraph input format. On external Epitope3D test set (45 antigens), PatchEpi consistently outperformed EpiGraph across all evaluated metrics after retraining. As shown in Table 2, PatchEpi more than doubled the MCC increasing from 0.10 to 0.26, and improved AUPRC from 0.09 to 0.25. The performance improvement of PatchEpi can be attributed to substantial differences in scale and label composition between the two datasets. The PatchEpi training set contained 2 124 761 residues, compared with 61 681 residues in the EpiGraph training set, and showed a longer median sequence length but lower mean epitope fraction than the EpiGraph training set (338 versus 254 aa; 8.87% versus 11.21%). We further compared PatchEpi with retrained EpiGraph on the leakage-controlled ANABAG test set, using the same ANABAG training and test partitions. The dataset was partitioned at 60% sequence identity, with residual train–test overlap assessed using ≥80% query and target coverage. PatchEpi achieved a 37.5% relative gain in MCC over EpiGraph, increasing from 0.24 to 0.33. The model also improved F1 from 0.26 to 0.30, AUC from 0.83 to 0.90, and AUPRC from 0.24 to 0.25 (Table 2).

**Table 2:** Performance comparison between retrained EpiGraph and PatchEpi on the Epitope3D blind test set (45 antigens) and the leakage-controlled ANABAG internal test set.

Dataset	Model	MCC	AUPRC	F1	AUC
Epitope3D blind test set	EpiGraph	0.10	0.09	0.14	0.64
Epitope3D blind test set	PatchEpi	**0.26**	**0.25**	**0.30**	**0.79**
ANABAG internal test set	EpiGraph	0.24	0.24	0.26	0.83
ANABAG internal test set	PatchEpi	**0.33**	**0.25**	**0.30**	**0.90**

Bold values indicate the better performance.

Cluster-level analysis further showed that PatchEpi retained predictive signal on 436 SARS-CoV-2 full-spike antigens (MCC = 0.33; recall = 0.72), despite their substantially longer length and lower epitope fraction than SARS-CoV-2-related training antigens (Supplementary Note 3 and Figs. S2-S3). Additionally, PatchEpi showed comparable performance on experimentally determined and AlphaFold-predicted antigen structures, supporting its applicability when experimental structures are unavailable (Supplementary Note 4 and Table S1).

### 3.2 Data leakage from similar antigen sequences

Conventional data partitioning strategies, particularly random splitting, are inherently susceptible to data leakage. Previous benchmarking work by Cia *et al*. (2023), also emphasized the need for redundancy-controlled evaluation in conformational B-cell epitope prediction, using sequence-identity clustering and representative selection to reduce overlap in benchmark construction. However, selecting a single representative from each cluster inevitably discards biologically relevant sequence variation.

In contrast, we retained all sequences within biologically defined clusters and split the dataset at the cluster level. By assigning each entire cluster to a single partition, we reduced the chance that highly similar or homologous antigens were distributed across training, validation, and test sets. Sequence-identity analysis showed that, among the 864 test antigens, only 29 (3.4%) had the closest training match with ≥90% sequence identity and ≥80% mutual coverage. In contrast, no leakage was detected in the Epitope3D blind test set of 45 antigens under the same procedure. A 60% identity threshold offered a balanced compromise between minimizing data leakage and preserving meaningful biological clustering and was adopted in subsequent analyses. Detailed comparisons across clustering thresholds and discussions of residual sequence overlap are provided in Note 1 and Fig. S1 of the Supplementary Materials.

**Figure 2:**
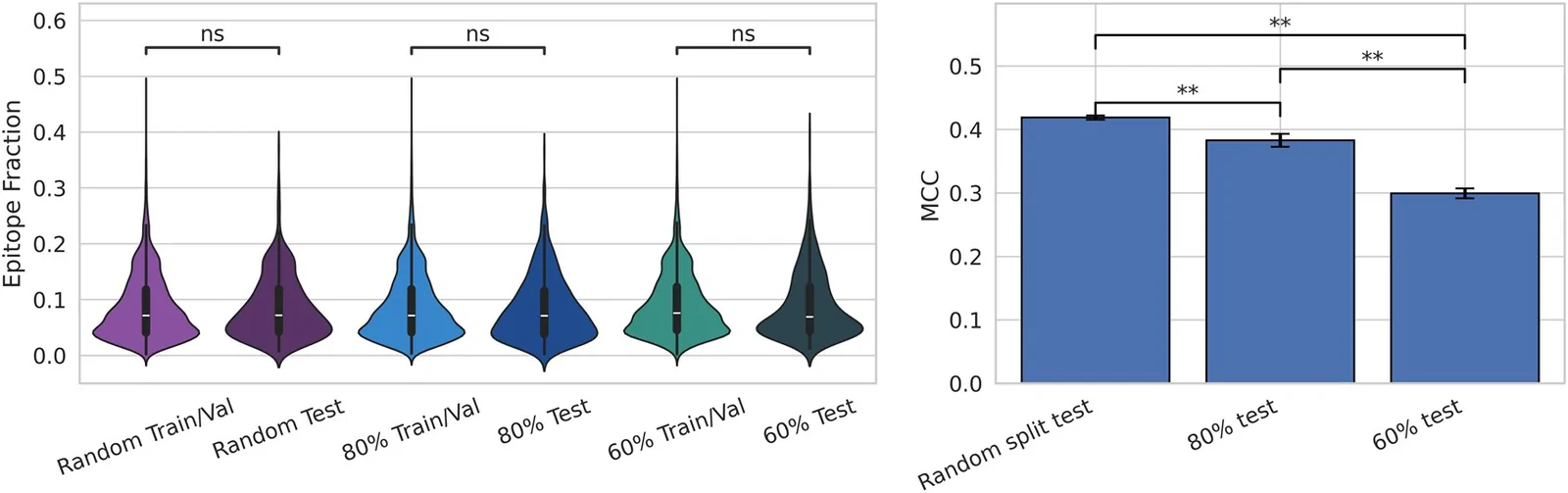
Comparison of epitope fraction (left) and model performance across different dataset splits. Left, epitope fraction; right, model performance measured by MCC. Results are shown for the non-clustered random split, the 80% sequence identity split, and the 60% sequence identity split. Statistical significance was assessed using a permutation test, with values indicated as ns (not significant) and ** (*P* < .01).

Furthermore, our model was trained and tested on three dataset partitioning conditions (no clustering, 80% sequence-identity clustering, and 60% sequence-identity clustering) to assess the impact of clustering stringency on model performance. As shown in Fig. 2, both the epitope fraction and model performance measured by MCC, were compared across splits. Statistical testing showed no significant difference in epitope fractions among the splits, suggesting that the performance drop was unlikely to be driven simply by differences in epitope-label density across test antigens. Under the random split, performance was highest because many test antigens were highly similar to the training set. Performance decreased at 80% sequence identity and dropped sharply at 60%, where train-test similarity was substantially reduced. This consistent decline in performance demonstrates that homology-controlled evaluations provide a more realistic assessment of model generalization than random splitting.

### 3.3 Patch-aware representation learning enables biologically realistic epitope modeling

**Figure 3:**
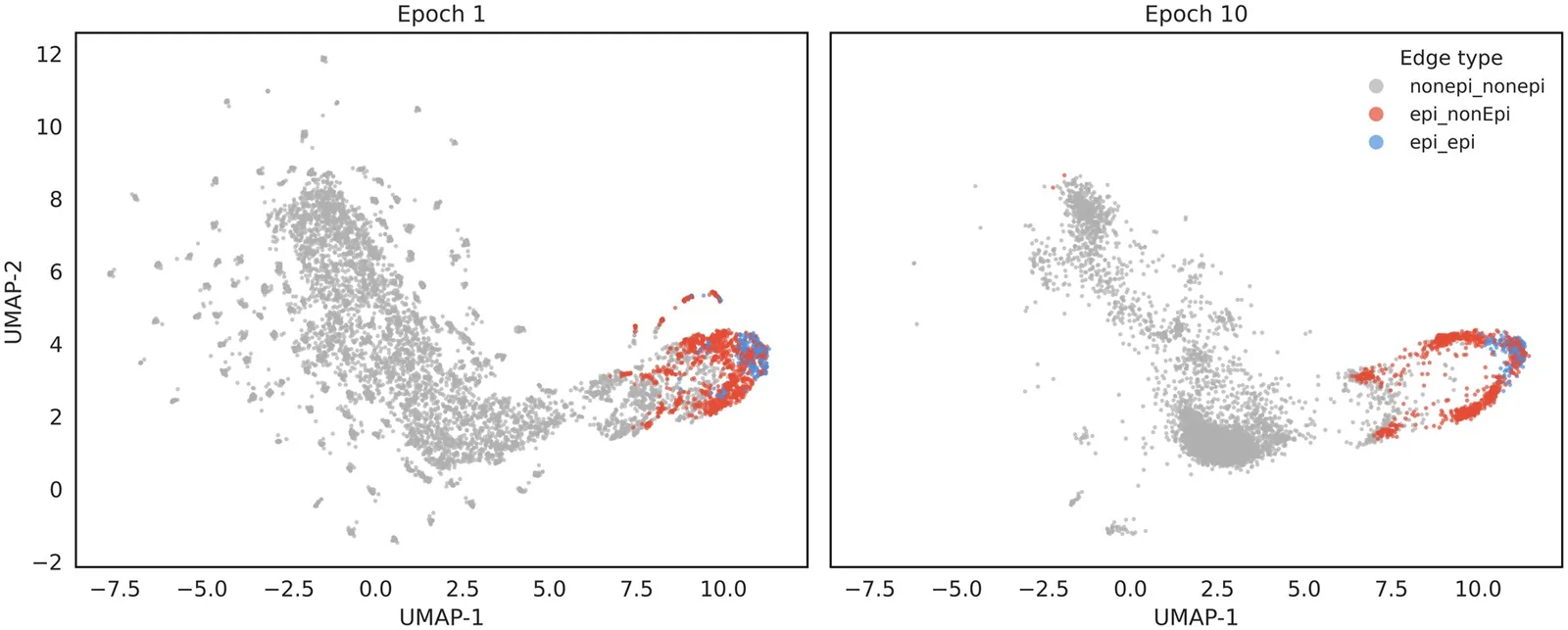
UMAP visualization of edge-level patch embeddings at early (epoch 1) and later (epoch 10) stages of training. As training progresses, epi–epi and nonepi–nonepi edges form more compact and separable regions, indicating that the model progressively organizes patch embeddings according to epitope relevance.

To better interpret the model behavior, we extracted the latent embeddings produced by the Patch-Coherence Encoder together with the edge-level attention features from the EGNN for downstream analysis. These UMAP (Uniform Manifold Approximation and Projection) projections shown in Fig. 3 illustrated how the Patch-Coherence Encoder’s latent space evolved for a specific antigen (8J7Y) during training. At Epoch 1, the edge embeddings suggested that the initial latent representation did not yet clearly separate different edge types. By Epoch 10, the latent embedding space became more structured. The overall Silhouette score increased from 0.195 to 0.293 from Epoch 1 to Epoch 10.

**Figure 4:**
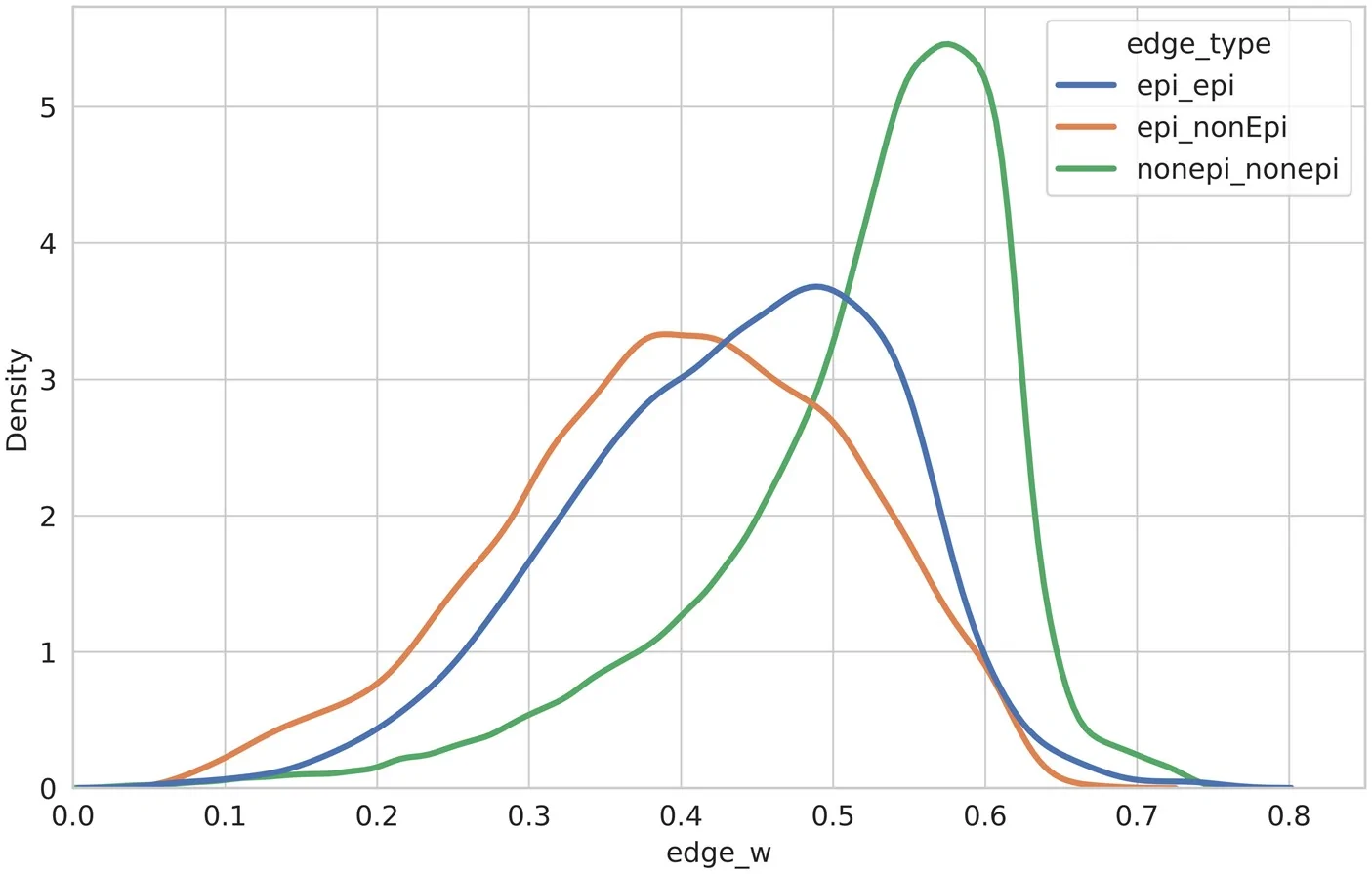
Density distribution of learned edge weights for epi–epi, epi–nonepi, and nonepi–nonepi edges. The edge weights differ across edge types, with nonepi–nonepi edges showing the highest weights and epi–nonepi edges showing the lowest weights.

In addition, the edge-attention mechanism modulates message passing in EGNN by assigning high, intermediate and low edge weights to nonepi–nonepi, epi–epi and epi–nonepi edges. The distribution of learned edge weights (edge_w) shown in Fig. 4 demonstrates that the edge-attention mechanism successfully captured biologically meaningful relationships. Nonepi–nonepi edges received the highest edge weights, with a median of 0.54, followed by epi–epi edges with a median of 0.44 and epi–nonepi edges with a median of 0.40. Edge-weight distributions differed significantly across the three edge types according to the Kruskal–Wallis test (*H* = 18 972 *P* < .001). Pairwise Mann–Whitney *U* tests confirmed significant differences between all three edge-type pairs. Nonepi–nonepi edges had significantly higher weights than epi–epi edges (*n* = 168 436 versus 3718; *U* = 4.6 × 10^8^; *P* < .001) and epi–nonepi edges (*n* = 168 436 versus 18 244; *U* = 2.4 × 10^9^; *P* < .001). Epi–epi edges also had significantly higher weights than epi–nonepi edges (*n* = 3718 versus 18 244; *U* = 4.0 × 10^7^; *P* < .001). However, this separation remains suboptimal as explained in Supplementary Note 7.

### 3.4 Ablation experiment and fine-tuning strategy

**Figure 5:**
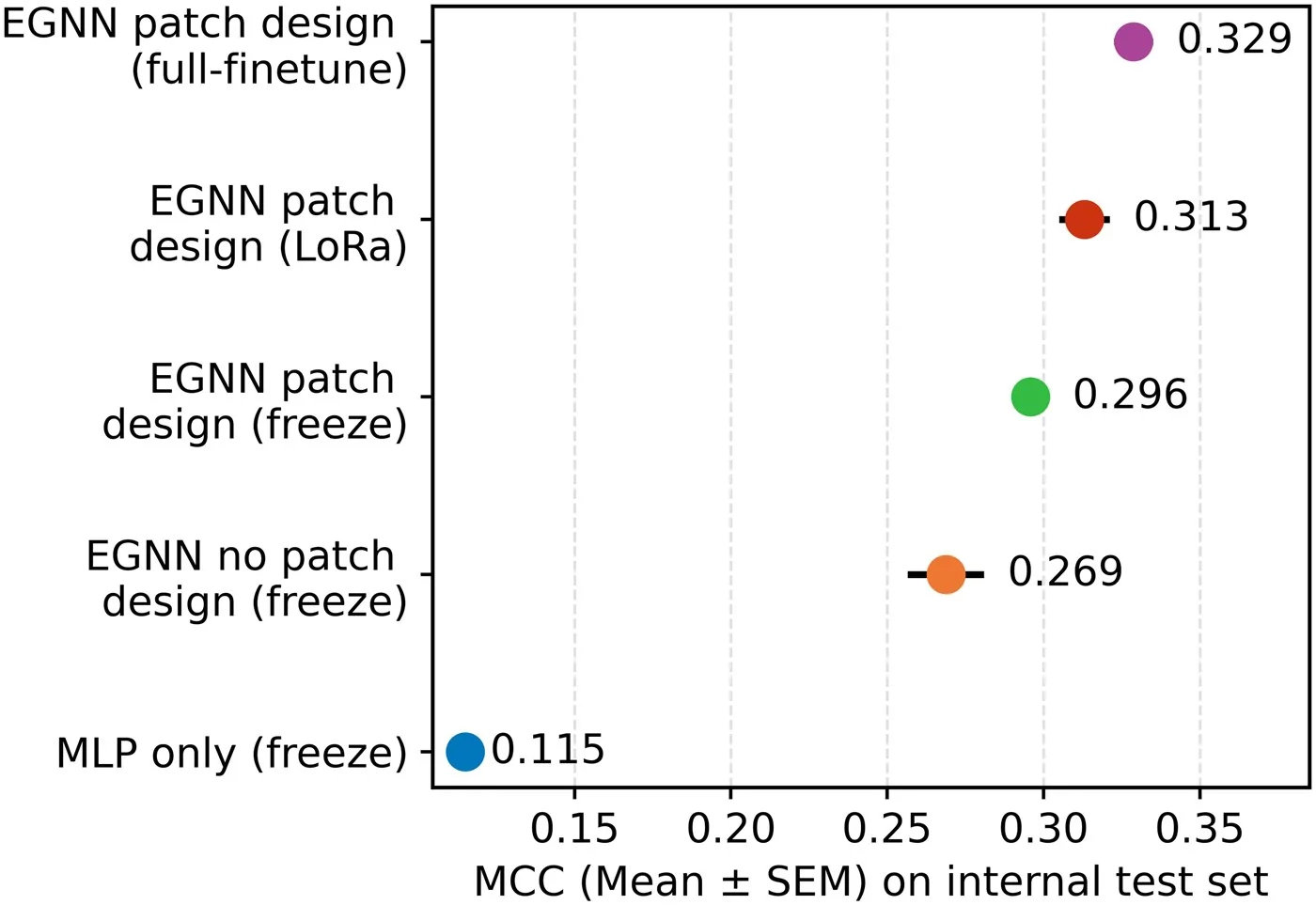
Ablation study comparing different architectural and training configurations with ESM2 as backbone. Unless otherwise indicated, models use the ESM2-150M backbone; the ESM2-650M result shows an additional backbone-size ablation. Patch-aware EGNN improves over no-patch EGNN and the MLP baseline, with full fine-tuning achieving the highest MCC. Points show mean MCC across three runs; error bars denote SEM.

Figure 5 illustrated the impact of incorporating structural information with ESM2 as the backbone under different training strategies on internal test set performance. The sequence-only baseline (ESM2 + MLP) achieved a mean MCC of only 0.115, confirming that sequence signatures alone are insufficient for identifying structural epitopes. Incorporating geometric reasoning through the EGNN substantially improved performance. The residue-level model without patch design reached a mean MCC of 0.269, highlighting the value of modeling 3D spatial organization. Introducing the patch design yielded further gains, increasing the mean MCC to 0.296. Optimizing ESM2 provided additional improvement. While applying LoRA (Hu *et al.* 2022) to the last two ESM2 layers raised the MCC to 0.313, full fine-tuning of ESM2 delivered the strongest performance. The no-patch EGNN also improved after full fine-tuning, reaching an MCC of 0.311, but remained below the patch-aware full-finetuned model. In addition, the larger ESM2-650M backbone did not improve performance (MCC = 0.310), suggesting that the gain of PatchEpi is not simply due to increasing the ESM2 backbone size. Detailed discussion was provided in Supplementary Note 5.

### 3.5 Case study

**Figure 6:**
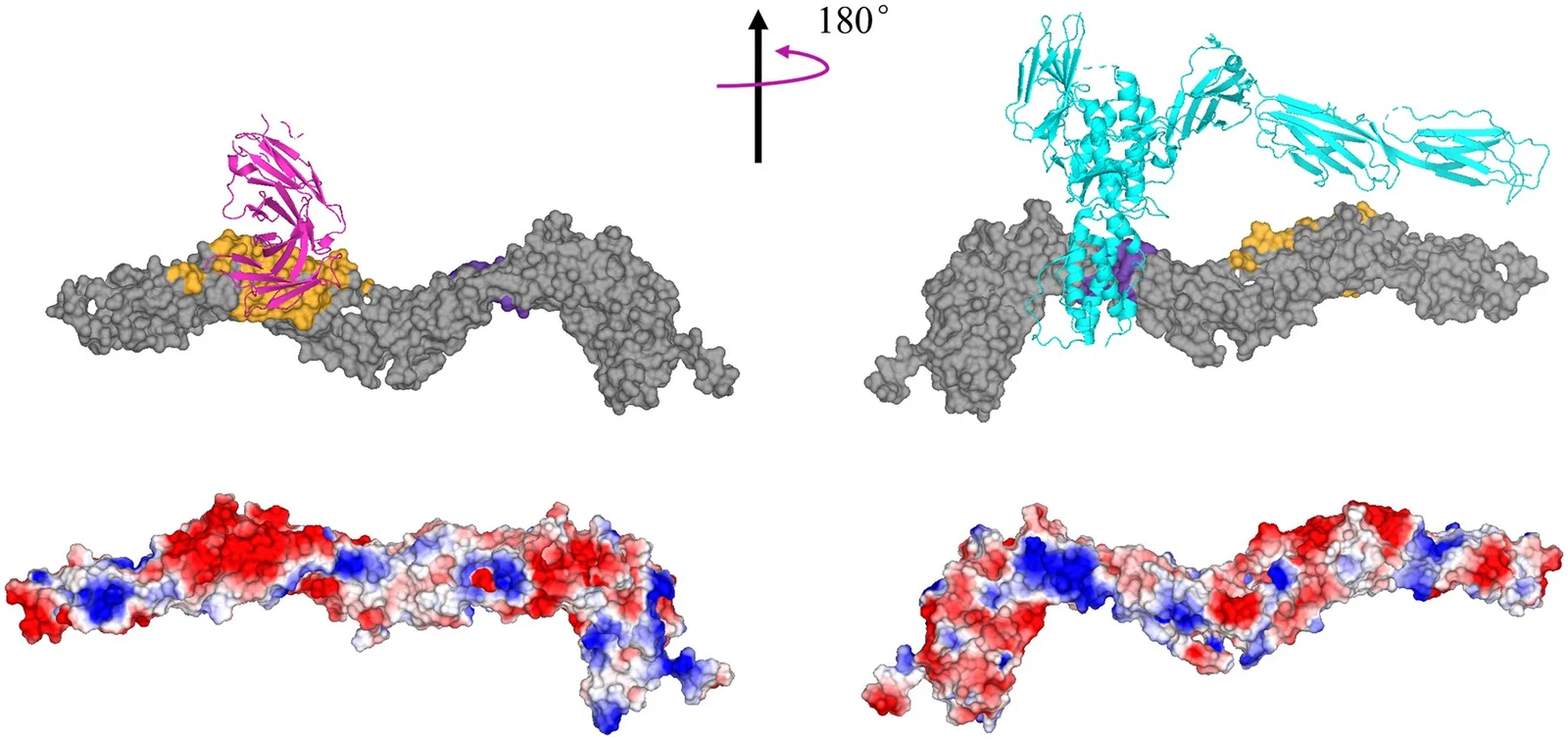
Patch-level predictions highlight chemically coherent surface regions for antigen 2EC8 in Epitope3D blind test set. Top panels show predicted surface patches (gold and purple) on the antigen in 4K9E and 2E9W respectively. In the left example, the predicted patch overlaps the antibody–antigen interface (antibody in magenta), while in the right example the patch corresponds to a PPI (partner in cyan). Bottom panels show the electrostatic potential mapped onto the same surfaces (red, negative; blue, positive), indicating that both patches reside in locally coherent physicochemical environments.

To illustrate the impact of patch design and the model’s performance on the Epitope3D blind test set, we presented the prediction results for Mast/stem cell growth factor receptor (PDB ID: 2EC8) in Fig. 6. The model achieved strong agreement with the reference epitope for the 2EC8 target (MCC = 0.767, AUC = 0.994), capturing the dominant surface footprint recognized by the antibody. As shown in the upper-left panel of Fig. 6, the experimentally determined epitope in the 4K9E crystal structure closely overlapped with the gold predicted surface patch, while the additional purple patch corresponded well to a protein–protein interaction (PPI) interface observed in PDB 2E9W, as shown in the upper-right panel. Structural analyses of 4K9E and 2E9W provided further support for the hypothesis that apparent false-positive predictions may correspond to alternative, biologically relevant PPI interfaces. Furthermore, the two predicted patches occupied regions with different electrostatic environments. The gold patch aligned with a predominantly negatively charged surface, while the other (purple) lay on a neutral and weakly polar region, near to a cluster of positively charged residues. Consequently, these patches correspond to different binding patterns, each of which is optimized for distinct physicochemical micro-environments on the antigen surface. The model’s ability to separate these regions highlights its capacity to encode and recognize fine-grained physicochemical determinants of antibody binding. This supports the initial idea of patch design.

**Figure 7:**
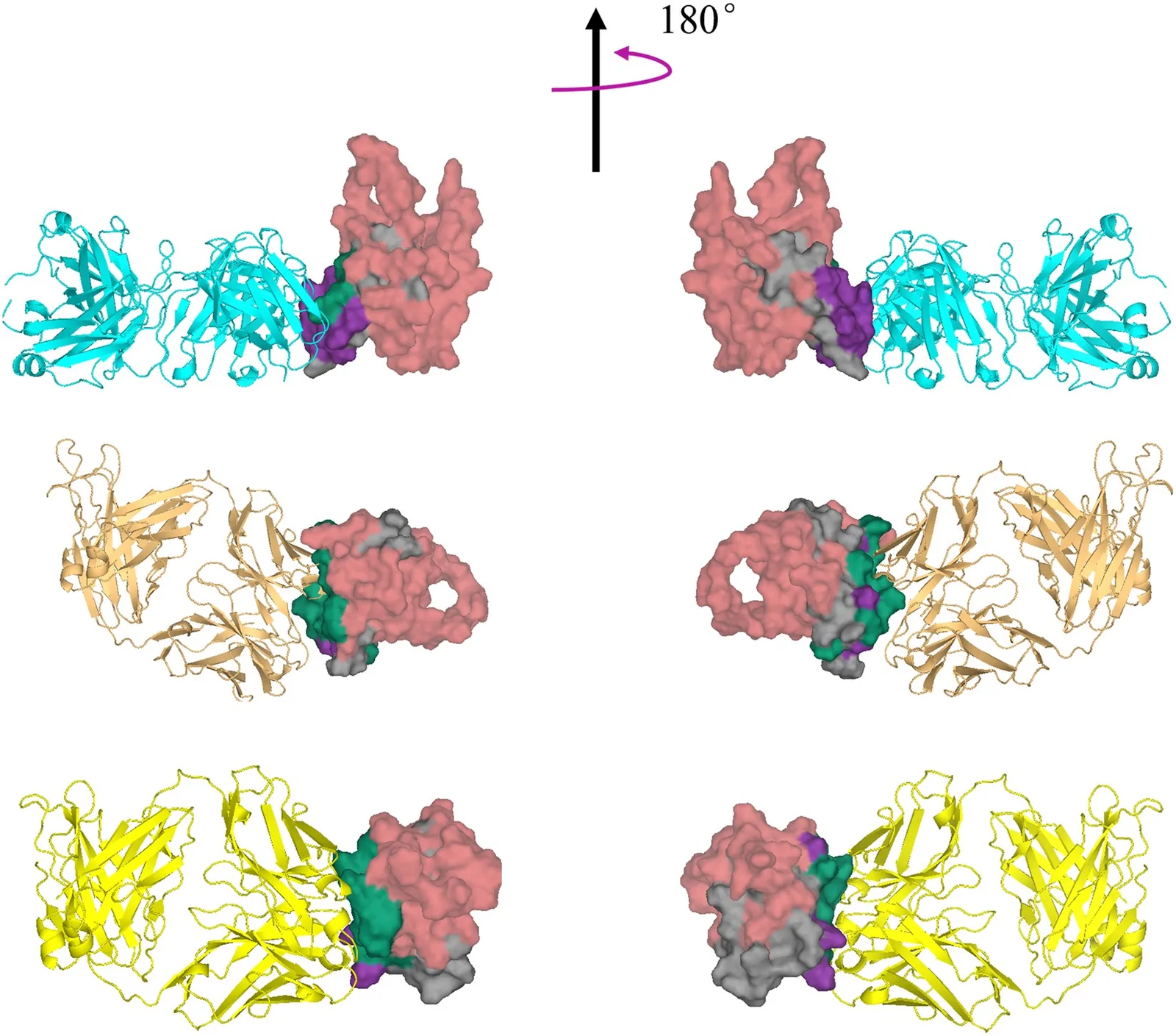
Residue-level comparison of epitope predictions for three nearly identical antigens bound to different antibodies. Top: 4PS4 (MCC = –0.3947, AUC = 0.130). Middle: 5L6Y (MCC = –0.079, AUC = 0.472). Bottom: 7REW (MCC = 0.195, AUC = 0.699). Surfaces are colored according to residue-level classification: missed true epitopes (purple), predicted epitopes (green), non-epitope residues (red), and ambiguous or partially buried residues (grey).

To delineate the operational boundaries of our model, we conducted a detailed analysis of outliers exhibiting suboptimal performance. A similar trend of underperformance was observed for PDB entries 4PS4 (MCC = −0.395, AUC = 0.130), 5L6Y (MCC = −0.079, AUC = 0.472), and 7REW (MCC = 0.195, AUC = 0.699), which correspond to three nearly identical antigens bound by different antibodies. Surface visualization (Fig. 7) and residue-level comparisons indicated that residues labeled as false positives in each complex frequently align with alternative antibody binding regions observed in other structures within the same cluster. Notably, all antigens in this group correspond to Interleukin-13 (IL-13), a small, soluble cytokine that mediates immune signaling by engaging multiple protein partners through exposed and flexible surface regions (Junttila 2018, Jaén *et al.* 2022). Given that such cytokines often harbor multiple, topographically distinct yet partially overlapping antibody-binding sites (Ultsch *et al.* 2013, Popovic *et al.* 2017), the prediction of multiple coherent surface regions is biologically plausible.

**Figure 8:**
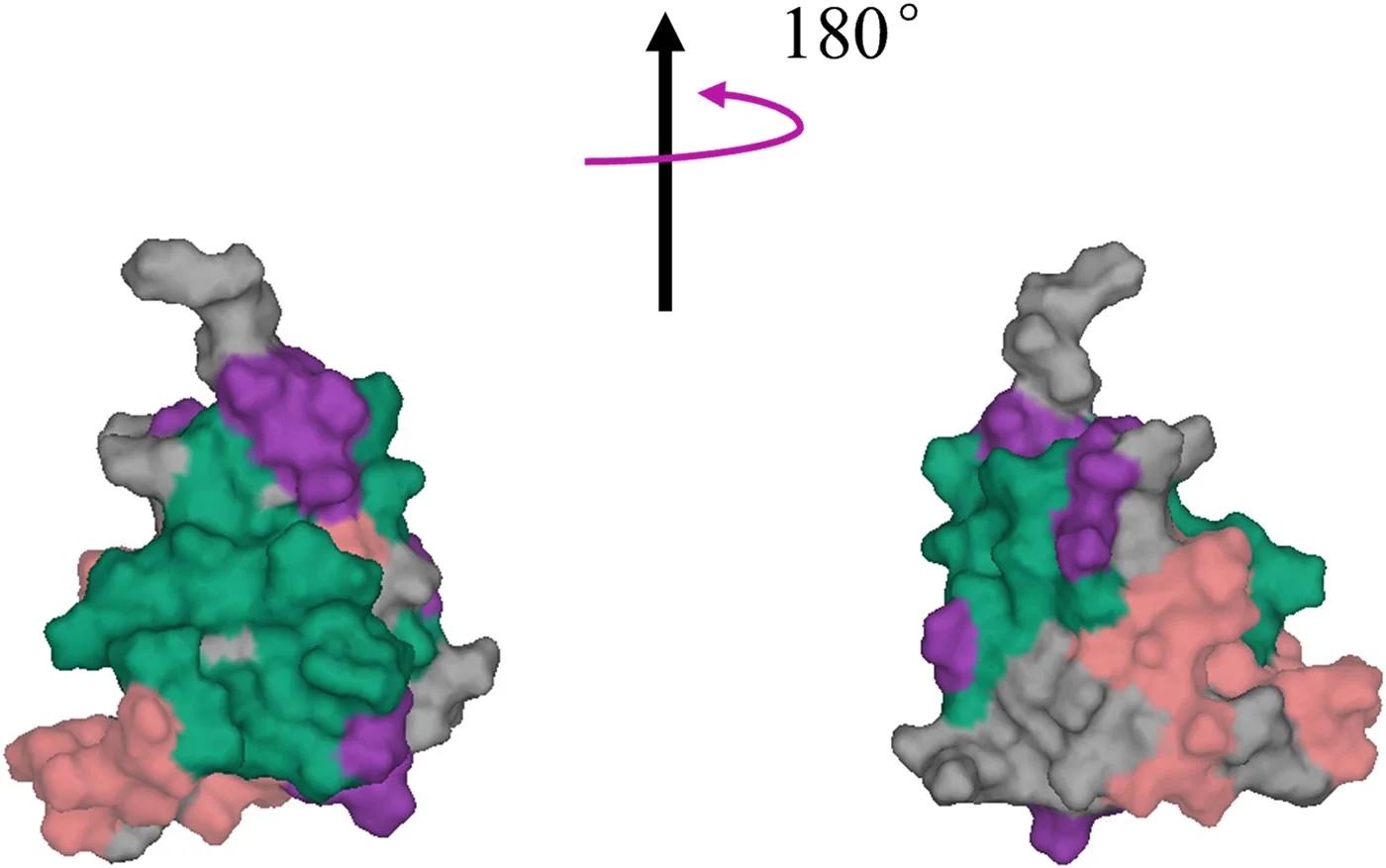
Model performance on antibody-derived and apo antigen structures. To determine whether predicted epitope regions reflect intrinsic antigen properties rather than residual bound-state geometry, we evaluated the model on the apo structure of IL-13 (1GA3). The model retains reasonable predictive performance (MCC = 0.225), indicating that the identified surface regions are not solely dependent on antibody-bound conformations.

In addition, it should be noted that the antigen structures used in these analyses are derived from antibody-antigen complexes by removing the antibody followed by Rosetta (Alford *et al.* 2017) relaxation; the residual features of the bound-state geometry may therefore persist. To further assess whether the predicted results reflect intrinsic antigen properties, we additionally examined the corresponding apo structure (1GA3) of IL-13. Notably, the model retained reasonable predictive performance in this setting (MCC = 0.225) as shown in Fig. 8, supporting the view that the identified surface regions are not solely dependent on antibody-induced conformations.

Additional case studies further illustrate the biological interpretation of PatchEpi predictions. For the structurally similar gp160–Fab complexes 4J6R and 4YDI, PatchEpi identified coherent antibody-binding patches with strong predictive performance. For nerve growth factor (NGF) in 6FFY, several apparent false positives overlapped with experimentally characterized protein–protein interaction interfaces, suggesting that some predicted regions reflect broader interaction-prone surfaces. Detailed analyses and structural visualizations are provided in Supplementary Note 6 and Figs. S4–S5.

## 4 Conclusion

In this work, we introduce a patch-aware framework for structure-based epitope prediction that reflects the biophysical principles of antibody recognition. By integrating ESM2 embeddings with an equivariant graph neural network and a patch encoder, the model captures local geometric context, enforces spatial coherence within epitope regions, and preserves sharp interface boundaries. Evaluated on a leakage-controlled split of ANABAG and the Epitope3D blind test set, the proposed model consistently outperforms existing state-of-the-art methods across all major metrics, including MCC, AUPRC and ROC-AUC. The framework scales robustly to large and structurally complex antigens, including long viral glycoproteins such as the SARS-CoV-2 spike, and generalizes effectively across heterogeneous antigen clusters. Overall, our study demonstrates that modeling epitope topology provides both performance and interpretability gains, and motivates future benchmarks that incorporate patch-level and context-aware epitope annotations.

## Supplementary Material

btag678_Supplementary_Data

## Data Availability

Data used in this study were obtained from publicly available resources, including ANABAG (https://github.com/DSIMB/anabag-handler) and Epitope3D (https://biosig.lab.uq.edu.au/epitope3d/data). The code and the standalone executable binary can be accessed from GitHub (https://github.com/wsicheng739/PatchEpi) and Zenodo (https://doi.org/10.5281/zenodo.20366444).
