# Supplementary material for: PatchEpi: patch-aware equivariant learning improves structure-based epitope prediction
Source: Bioinformatics. 2026 Sep 15;42(9):btag678. doi: 10.1093/bioinformatics/btag678 (PMC13613061; doi:10.1093/bioinformatics/btag678)
Supplement: Supplementary file 1 [file btag678_supplementary_data.zip › supplementary information.docx]

# SUPPLEMENTARY MATERIALS

# Notes

## 1. Dataset Partitioning and Data Leakage

Most existing structure-based epitope prediction studies rely on pairwise sequence-identity filtering to prevent train-test redundancy [1,2]. This approach avoids the algorithmic complications of clustering, such as representative selection and order dependence, and is straightforward to implement. However, pairwise identity filtering treats each protein pair independently and does not encourage any higher-level biological grouping on the dataset. By contrast, clustering aims to group antigens into families, with the goal of ensuring that antigens belonging to the same biological or structural lineage are not split across training and testing sets. In principle, this strategy produces partitions that better reflect biological independence, since antigens within the same family may present structurally similar epitopes despite their global sequence divergence. This is relevant for antibodies, where epitope formation depends on local surface geometry and domain context rather than overall sequence similarity.

However, clustering itself introduces additional challenges. MMseqs2 clustering is based on heuristic, representative-based procedures rather than exhaustive pairwise exclusion. In the default setting used here, MMseqs2 applies a greedy set-cover strategy, where cluster assignment depends on the selected representatives, local alignment relationships, coverage filtering, and sequence composition [3–5]. Therefore, cluster-level partitioning does not guarantee that every train–test sequence pair below a desired redundancy threshold has been removed. In particular, PDB-derived antigen sequences may differ in length because of truncations, unresolved residues, engineered constructs, missing termini, or domain boundaries. As a result, two antigens can remain in different clusters despite sharing high sequence identity over a biologically relevant region.

To quantify the extent of potential data leakage associated with cluster-based splitting strategies, we analyzed the cumulative percentage of test sequences exhibiting identity to the training set under varying sequence-identity thresholds (50%, 60%, 70%, 80%, 90%, and no clustering), considering only alignments that satisfied the ≥80% bidirectional coverage criterion (Figure S1). Here, cumulative percentage means that, for example, the ≥60% identity value also includes test sequences with training-set hits at ≥70% and ≥90% identity. Although stricter clustering can reduce train–test similarity, overly stringent thresholds such as 50% can over-cluster biologically distinct antigens. G proteins (8HPT, 8HQC, and 8HS3), for example, were clustered into a single GPCR cluster. A 60% identity threshold therefore offered a balanced compromise between minimizing data leakage and preserving meaningful biological clustering and was adopted in subsequent analyses.

**Figure S1. Effect of MMseqs2 clustering threshold on residual train–test sequence overlap.** For each dataset split, test sequences were searched against the training set using MMseqs2. The y-axis shows the percentage of test sequences with at least one training-set hit meeting or exceeding the indicated sequence identity threshold, with bidirectional coverage ≥80%. Accumulative percentage means that the ≥60% identity value also includes test sequences with training-set hits at ≥70% and ≥90% identity. Cluster-50 to Cluster-90 indicate dataset splits generated using MMseqs2 clustering at 50–90% sequence identity, whereas “No clustering” represents a non-clustered split.

Given these limitations, we retained MMseqs2 clustering to preserve antigen-family-level structure, but additionally performed a post-clustering all-to-all identity check to quantify residual high-similarity train–test relationships. In the ANABAG-derived benchmark, residual high-similarity relationships were rare. Among the 864 test antigens, only 29 (3.4%) had a closest training match with ≥ 90% sequence identity and ≥ 80% mutual coverage, and the cumulative proportions at the ≥ 80% and ≥70% identity thresholds remained low, at 4.1% and 4.9%, respectively. In contrast, no leakage was detected in the independent Epitope3D blind test set of 45 antigens under the same post-hoc identity-checking procedure. We did not remove the residual high-similarity cases from the ANABAG-derived benchmark because they represented only a small fraction of the dataset and their removal would reduce antigenic diversity. Instead, we annotated these cases as potential homology-level leakage and retrained EpiGraph using the same ANABAG-derived partitions as PatchEpi. Therefore, both models were evaluated under the same residual-similarity regime on the ANABAG-derived benchmark, while the Epitope3D blind test set provided an additional leakage-free evaluation.

## 2. Benchmark datasets and their use in model training and evaluation

Diverse benchmark datasets are essential for the development and evaluation of structure-based B-cell epitope predictors that generalize beyond a narrow set of antigen families. In practice, however, assembling diverse antibody–antigen datasets remains challenging due to the limited availability of experimentally resolved complexes.

The Epitope3D dataset has become a commonly used benchmark for evaluating structure-based B-cell epitope predictors. Its construction involves transferring antibody-antigen interface annotations from experimentally resolved complexes to related unbound structures identified through sequence- or structure-based similarity searches [6]. While this strategy expands dataset coverage, it also introduces antigens whose biological context may not naturally support antibody recognition. For example, in some cases the epitope-bearing chain originates from a naturally oligomeric or pore-forming complex (e.g., 2X53 and 5ZDH), yet Epitope3D includes only the isolated chain rather than the full quaternary assembly. As a result, residues that are buried in the native multimer may appear artificially exposed. Moreover, certain antigens included to maximize structural diversity may be less representative of antibody–antigen systems commonly encountered in experimental or therapeutic studies. Therefore, direct training on the Epitope3D collection may introduce biologically unrealistic surface patterns, potentially limiting model generalization to relevant antibody-recognition scenarios. In contrast, databases such as SAbDab [7] and ANABAG partially mitigate these issues by retaining experimentally resolved antibody–antigen complexes, thereby preserving realistic antibody accessibility. However, they do not provide standardized antigen-family or functional annotations. In this work, we adopt a complementary strategy. Rather than training on the Epitope3D training set, we rely exclusively on the ANABAG dataset. Model evaluation is then performed on the Epitope3D blind test set, whose curation and widespread use in the community ensure comparability with existing structure-based predictors.

A more robust direction for future work might be designing datasets and benchmarks that explicitly consider antigen family, organism category, and structural domain. Such efforts could involve organizing antigens according to viral, bacterial, or eukaryotic origin, as well as grouping structurally coherent families such as GPCRs, coronavirus spikes, and immunoglobulin-like domains. In addition, structural datasets may be annotated with external metadata from UniProt (https://www.uniprot.org/) or IEDB’s (https://www.iedb.org/) antigen records, enabling organism identity to be incorporated during training or used to construct biologically meaningful evaluation sets. As of June 2026, SAbDab2 has been released as a machine-learning-oriented extension of SAbDab, providing systematically annotated antibody structures, antibody–antigen complexes, and leakage-controlled benchmark splits for model development and evaluation [8].

## 3. Analysis of SARS-CoV-2 Full-Spike in ANABAG test set

Leveraging MMseqs2 clustering during data processing, Figure S2 summarized model behavior across antigen families by plotting the mean MCC against the number of antigens within each cluster. Most clusters exhibited positive MCC values, tightly concentrated in the 0.1–0.3 range for small to medium cluster sizes. The SARS-CoV-2 full-spike cluster in the test set contained 436 longer antigens and a much lower mean epitope fraction than 616 SARS-CoV-2-monomer antigens represented in training (1099.4 amino acids and 2.1% vs. 198.6 amino acids and 12.6%; see Figure S3). This represents a particularly challenging setting because the lower epitope fraction creates a stronger imbalance between epitope and non-epitope residues, making predictions more sensitive to false positives. This difficulty is directly supported by Cia et al., who used the SARS-CoV-2 spike trimer as an independent case study and showed that conformational B-cell epitope predictors generally struggled on this large antigen [9].

We further assessed whether the model could make predictions on SARS-CoV-2 full-spike antigens after being trained with shorter SARS-CoV-2-related antigens. Under this setting, the model achieved an MCC of 0.33 and recall of 0.72 on the full-spike cluster, suggesting that it retained predictive signal when applied to substantially longer, closely related SARS-CoV-2 antigen structures. To assess potential leakage, we performed an MMseqs2 identity check between the 436 full-spike sequences and the training set using sequence identity ≥ 60%, query coverage ≥ 80%, and target coverage ≥ 80% as leakage criteria. No full-spike sequence satisfied these leakage-level criteria against the training set. Therefore, we interpret the SARS-CoV-2 full-spike cluster as a challenging within-family extrapolation case.

Figure S2. Cluster-level performance distribution across the ANABAG internal test set. Each point represents a homologous antigen cluster, with marker size proportional to cluster size and color denoting mean MCC. Annotated numbers indicate the MMseqs2 cluster IDs assigned during dataset splitting. The SARS-CoV-2 spike cluster appears as a large, high-performing outlier, whereas most smaller clusters exhibit broader variability in prediction accuracy.

**Figure S3**. Length distribution shifts between training set and SARS-Full-Spike cluster. Due to such a large shift, SARS-Full-Spike cluster was assigned in ANABAG internal test set.

## 4. Robustness of PatchEpi to AlphaFold-predicted antigen structures

Because the DiscoTope 3.0 data were not included during ANABAG/Epitope3D preprocessing, clustering, or split construction, sequence- or structure-level redundancy may exist between the training data and the DiscoTope antigens. This introduced data leakage, meaning that absolute values of MCC, AUC, and AUPRC on DiscoTope 3.0 cannot be interpreted as true measures of generalization performance. Nevertheless, we included this evaluation to assess compatibility with AlphaFold-predicted structures [10]. DiscoTope provides both experimentally solved and AlphaFold2-predicted models, enabling us to test whether PatchEpi maintains stable predictions under structural noise introduced by AlphaFold2. In Table S1, the small performance gap observed between solved and predicted structures indicates that the model’s patch-aware reasoning is robust to coordinate perturbations, demonstrating strong practical usability even when only AlphaFold structures are available. This stability may also reflect that many epitope residues are located in ordered surface regions, where AlphaFold2 models tend to preserve the overall local geometry better than in flexible or disordered regions [11].

Table S1. Comparison of epitope prediction performance when using experimental (solved) antigen structures versus AlphaFold-predicted monomers. The absolute values may be inflated due to data leakage in the DiscoTope 3.0 set.

| Structure Source | MCC | AUPRC | F1 | AUC |
| --- | --- | --- | --- | --- |
| Solved | **0.60** | 0.69 | **0.59** | 0.94 |
| AlphaFold predicted | 0.59 | 0.69 | 0.58 | 0.94 |

## 5. Importance of model components and fine-tune strategies

PatchEpi integrates three complementary components, including an ESM2 sequence encoder, a graph autoencoder–based patch coherence module, and an EGNN for geometry-aware message passing. Recent epitope prediction methods increasingly employ pretrained protein language models to capture residue-level biochemical and evolutionary context. Protein-specific BERT models, such as ProteinBERT, learn biologically meaningful sequence features from relatively smaller datasets, and achieve reasonable results on linear epitope prediction [12,13]. ESM-2 extends this paradigm by scaling masked language modeling to hundreds of millions of protein sequences. In our framework, we adopt ESM2-150M rather than larger variants, as increased encoder capacity does not necessarily benefit patch-level epitope prediction. Indeed, over-confident residue-level representations can dominate geometric aggregation and hinder the learning of spatially coherent surface patches, leading to fragmented false-positive predictions.

Linear epitope prediction models primarily relied on sequence-based deep learning architectures, including convolutional neural networks (CNNs) and recurrent neural networks such as long short-term memory (LSTM) models [14–17]. While these deep learning approaches can achieve reasonable results, they inherently lack three-dimensional context and are therefore limited in their ability to model conformational epitopes. Graph neural networks, including GCNs and related variants, enable information propagation across conformational neighborhoods and have demonstrated improved performance over sequence-only approaches [18]. However, conventional GNN-based approaches encode spatial relationships through graph topology without guaranteeing to produce consistent predictions under rotations or translation of the entire antigen [19]. This limits their ability to capture the coherent surface geometry of epitopes, often resulting in scattered predictions. We address this limitation by using an EGNN with equivariant message passing, where inter-residue distances modulate feature updates, thereby preserving geometric consistency and promoting coherent surface-level predictions.

The improved performance of PatchEpi can be attributed to its balance between residue-level discrimination and patch-level coherence. Sequence embeddings from ESM2 provide informative and moderated epitope signals, while geometric locality constraints restrict aggregation to physically plausible neighborhoods. Patch-level representations, combined with message passing weighted by patch similarity, encourage consistent predictions among spatially adjacent residues, enabling epitope signals to consolidate into contiguous surface regions. In addition, boundary loss, acting as a regulatory mechanism, suppresses predictions at patch margins, thereby reducing fragmented false positives commonly observed in residue-wise classifiers. Collectively, these components align the learning objective with the biological reality that antibody recognition occurs over coherent three-dimensional surface patches, accounting for the observed improvements in spatial consistency across diverse antigens.

Consistent with this interpretation, the comparison in Figure S4 demonstrates that residue-level classification is fundamentally limited. Substantial gains emerge only when the model incorporates information at the level of 3D surface regions rather than isolated residues. Introducing patch-level inductive bias further aligns the model with the biological reality that antibodies recognize continuous surface patches. The comparison between Low-rank adaptation (LoRA) with full-finetuning highlights the critical role of the underlying representation learned from protein language modeling in epitope prediction. LoRA provides a lightweight mechanism for adapting the upper layers of ESM2, yielding moderate performance gains [20]. However, full-finetuning achieved the best performance, suggesting that deeper representational shifts are necessary to optimally capture the biochemical and geometric cues associated with antibody–antigen interfaces [21,22].

The patch design also plays a central role in shifting the pretrained ESM2 representation from sequence-centric reasoning toward the surface-level organization that governs antibody binding. While ESM2 encodes contextual biochemical patterns along the primary sequence, it lacks an inherent notion of surface continuity or 3D patch formation. By grouping residues into spatial patches and enforcing consistent predictions within each patch, the model is encouraged to reorganize ESM2’s sequence-derived features around geometric neighborhoods. This process redirects the pretrained knowledge toward the epitope patches that antibodies recognize, enabling the network to capture the cooperative nature of epitope formation that are inaccessible to purely residue-level prediction approaches.

## 6. Case study

To illustrate different aspects of PatchEpi behavior, including successful epitope localization, apo-structure-based prediction, difficult prediction scenarios, and biologically relevant interface-level predictions, we summarized the analyzed antigens, data sources, leakage-level hit status, and model performance in Table S2. The selected cases include Envelope glycoprotein gp160–Fab complexes, which were used to assess PatchEpi’s ability to localize antibody-binding regions on viral surface antigens; IL-13 antibody-bound and apo structures, which enabled evaluation of prediction consistency; nerve growth factor examples, which represent challenging cases for examining potential failure modes; and mast/stem cell growth factor receptor, which was included to explore possible overlap between predicted epitopes and broader protein–protein interaction interfaces.

Table S2. Summary of case-study antigens, leakage status, and model performance.

| PDB ID | Sources | Target | Leakage level hit | MCC | | AUC | |
| --- | --- | --- | --- | --- | --- | --- | --- |
| 4J6R | ANABAG test set | Envelope glycoprotein gp160 | Has leakage^1^ | 0.779 | 0.989 | |  |
| 4YDI | ANABAG test set | Envelope glycoprotein gp160 | Has leakage | 0.759 | 0.991 | |  |
| 4PS4 | ANABAG test set | IL-13 | No leakage | -0.395 | 0.130 | |  |
| 5L6Y | ANABAG test set | IL-13 | No leakage | -0.079 | 0.472 | |  |
| 7REW | ANABAG test set | IL-13 | No leakage | 0.195 | 0.699 | |  |
| 1GA3 | Apo from PDB | IL-13 | N/A | 0.225 ^2^ | N/A | |  |
| 6FFY | Epitope3D blind test | Nerve growth factor | No leakage | -0.477 | 0.207 | |  |
| 1SGF | Protein-protein complex from PDB | Nerve growth factor | N/A | N/A | N/A | |  |
| 2EC8 | Epitope3D blind test | Mast/stem cell growth factor receptor | No leakage | 0.767 | 0.994 | |  |
| ^1^ Leakage-level hit was defined as an MMseqs2 best hit to the training set satisfying sequence identity ≥ 60%, query coverage ≥ 80%, and target coverage ≥ 80%. N/A indicates that the metric was not applicable for that case.  ^2^ For the apo IL-13 structure 1GA3, MCC was calculated using a combined reference epitope defined by aggregating epitope residues from all available antibody-bound IL-13 complexes. | | | | | | |  |

To qualitatively examine model behavior in high-performing antigen–antibody complexes, we examined the prediction on 4J6R and 4YDI, two Envelope glycoprotein gp160–Fab complexes, in which the antibody binds a compact surface epitope on the antigen. While the antigens are nearly identical, their structures exhibit subtle variations in atomic coordinates. The model achieved strong agreement with the experimental epitope in 4J6R (MCC = 0.779, AUC = 0.989) and 4YDI (MCC = 0.759, AUC = 0.991), capturing the dominant surface footprint recognized by the antibody. However, post-hoc all-to-all identity analysis showed these two cases have potential leakage to gp120 in 7LLK complex, with a maximum sequence identity of 79.4% to training-set antigens and high bidirectional coverage. Nevertheless, structural visualization, as shown in Figure S4, revealed that the predicted epitope formed a continuous, topologically coherent surface patch (green) that closely overlapped with the true binding interface. The antibody heavy-light variable domains positioned directly above this region, confirming that the model correctly identified the structural basin responsible for antibody engagement. A subset of residues at the edge of the binding site were assigned as false positives (red), but these residues were adjacent to the true interface and exhibited similar solvent exposure and curvature, suggesting that the model was responding to local geometric cues rather than producing arbitrary errors. Residue-level comparison reveals that the predicted epitopes from both structures are highly congruent, differing by only a single residue (Table S6). This finding aligns with the observation that these two antibodies occupy nearly identical epitope regions.

Figure S4. Epitope prediction on the 4J6R complex (top) and 4YDI (bottom). Surface view of both antigens highlights correctly predicted epitope residues (green), false positives (red), and missed true epitopes (purple). The predicted patch aligns closely with the antibody footprint (magenta and yellow), capturing the core epitope with minimal peripheral misclassification.

Inspired by previous observation, we further hypothesize that additional false positives observed in other antigens may reflect general protein-protein interfaces, rather than epitope specificity alone. To verify this hypothesis, we examined 6FFY, the worst-performing case in the Epitope3D blind test set (MCC = –0.477, AUC = 0.207), as shown in Figure S5. This structure corresponds to Nerve Growth Factor (NGF), a signaling protein that engages multiple binding partners through distinct exposed surface regions [23]. Under antibody-centric epitope annotations, many residues predicted by the model are therefore labeled as false positives. However, comparison with the 7S NGF complex (1SGF) reveals that a substantial fraction of these residues overlaps with experimentally validated PPI interfaces in multi-protein assemblies. Consistent with this interpretation, additional NGF complexes reported in the literature, including NGF bound to the p75 neurotrophin receptor [24] and the VPS10 domain–containing receptor SorCS2 [25] demonstrate that NGF utilizes multiple surface regions for biologically relevant protein–protein interactions. Also, multiple antibody–NGF complex structures (5JZ7, 9FIK, 4EDW and 4EDX) have also been resolved and exhibit distinct binding modes on these same antigen surfaces.

Figure S5. Predicted epitope-like regions on NGF overlap with protein–protein interaction interfaces on 1SGF. (Top): NGF structure colored by model predictions, with residues predicted as epitope-like shown in red and the interacting partner in blue. (Middle): Overlap between predicted regions and experimentally defined PPI interfaces. (Bottom): NGF in complex 1SGF with four binding patterns.

## 7. Limitations of current model

Although the model performs well across diverse antigen families, its accuracy remains constrained by several structural, methodological, and biological factors. A primary limitation lies in the dependence on complete and biologically accurate antigen structures. Since the model infers residue roles from solvent exposure, curvature, and spatial context, any deviation in the PDB input, such as missing loops or unresolved flexible regions, directly distorts the geometric features. In truncated structures, exposed surfaces may be inaccessible in the native conformation. Under these conditions, the model can highlight artificially exposed regions that resemble epitope-like patches despite having no biological relevance.

A second limitation arises from the absence of patch-level ground truth in existing epitope datasets. While the architecture is explicitly designed to capture coherent surface patches and interaction motifs, available annotations are limited to individual residues. This prohibits direct supervision on geometric continuity, boundary sharpness, and epitope extent. As a result, predictions that identify the shape or orientation of an epitope patch may still receive poor residue-level scores, whereas isolated residue predictions can achieve high scores despite lacking meaningful spatial coherence. This mismatch between model design and label granularity underestimates the model’s capacity to reason over surface patches and constrains our ability to assess error sources beyond single residues.

A related limitation concerns the incomplete separation of interactions across epitope boundaries. The edge-weight distribution for epi-nonepi overlaps with both epi-epi and nonepi-nonepi edges, suggesting that the model does not fully penalize cross-boundary interactions. This observation is consistent with case studies, where false-positive residues tend to accumulate near epitope boundaries. To achieve stronger boundary preservation, future optimization may require explicit constraints that further separate epi-nonepi edges from both homogeneous groups. Such refinements could sharpen functional boundaries between epitope and non-epitope regions and enhance the model’s ability to resolve subtle epitope boundaries on complex antigen surfaces.

The model-behavior analyses highlight the advantage of treating epitopes as surface patches. Patch-based predictions naturally align with coherent 3D binding surfaces, making them structurally interpretable and more readily integrated into downstream tasks such as docking or antibody design. This representation also motivates patch-level evaluation criteria. For example, assessing whether the dominant predicted patch spatially overlaps the true epitope region provides a biologically more consistent assessment than residue-wise metrics alone. In this way, epitope-patch modeling reframes the prediction problem to better reflect antigen–antibody interactions, where the functional unit is a continuous surface patch rather than a collection of independent residue labels.

Another important limitation arises when an antigen supports multiple valid antibody-binding modes. Because the model is antibody-agnostic, it cannot determine which binding patch is used by the specific antibody in the reference complex. Instead, it highlights all surfaces that are structurally plausible for antibody engagement. When only one of these patches is annotated in the dataset, the remaining unused epitopes are counted as false positives, creating the appearance of incorrect predictions even though the model is capturing antibody-compatible patterns. This is an unavoidable ambiguity when only the antigen is provided as input.

These factors delineate the principal failure modes of the current approach. Structural incompleteness distorts geometric features and residue-level annotations obscure patch-level correctness, when only antigen-only information is available. Addressing these limitations will be crucial for advancing beyond residue-wise scoring toward fully contextualized, interaction-aware epitope prediction.

# Additional Tables

Table S3. Detailed explanation of symbols in the equations

| **Symbol** | **Annotation** |
| --- | --- |
| $i,j$ | Residue indices within an antigen |
| $\mathcal{l}$ | EGNN layer index |
| $s_{i}$ | Amino acid at residue position |
| $h_{i}$ | Context-aware residue embedding from ESM2 |
| $x_{i}^{(\mathcal{l})}$ | Node feature of residue at EGNN layer |
| $r_{i}\in\mathbb{R}^{3}$ | 3D coordinate of residue |
| $\mathcal{E}_{\text{patch }}$ | Patch graph edge set defined by residue pairs within |
| $\mu_{i}$ | Mean of latent patch distribution for residue |
| $\sigma_{i}$ | Standard deviation of latent patch distribution |
| $z_{i}$ | Latent patch embedding of residue |
| $\tilde{z}_{i}$ | L2-normalized patch embedding |
| $\epsilon$ | Gaussian noise sampled from 𝒩(𝟎,𝐈) |
| $s_{ij}$ | Patch similarity between residues 𝑖 and 𝑗 (cosine similarity) |
| $\hat{s}_{ij}$ | Batch-normalized patch similarity |
| $\alpha$ | Learnable scalar controlling patch-similarity gating strength |
| $w_{ij}$ | Patch-similarity-based edge weight |
| $\mathcal{N(}i)$ | Set of spatial neighbors of residue 𝑖 |
| $\phi_{m}(\cdot)$ | Learnable EGNN message function (MLP) |
| $m_{ij}^{\mathcal{(l)}}$ | Message passed from residue 𝑗 to 𝑖 at layer ℓ |
| $y_{i}\in\{0,1\}$ | Ground-truth epitope label of residue 𝑖 |
| $\hat{y}_{i}\in[0,1]$ | Predicted epitope probability |
| $w_{1},w_{0}$ | Class-balancing weights for BCE loss |
| $\mathcal{E}$ | Residue–residue proximity edge set |
| $\mathcal{B}\subset\mathcal{E}$ | Set of neighboring residue pairs with opposing labels |
| $p_{ij}^{+}$ | Predicted probability for epitope-labeled residue in a boundary pair |
| $p_{ij}^{-}$ | Predicted probability for non-epitope residue in a boundary pair |
| $m$ | Margin parameter for boundary loss ( ) |
| $\lambda_{\text{BCE }}$ | Weight of binary cross-entropy loss |
| $\lambda_{\text{smooth }}$ | Weight of smoothness (Laplacian) loss |
| $\lambda_{\text{boundary }}$ | Weight of boundary contrastive loss |

**Table S4** Sensitivity analysis with alternative cutoffs of radius without retraining

| Test dataset | Radius | MCC | Precision | Recall |
| --- | --- | --- | --- | --- |
| Internal ANABAG test set | 6.0 | 0.330 | 0.197 | 0.693 |
| Internal ANABAG test set | 8.0 | 0.333 | 0.194 | 0.717 |
| Internal ANABAG test set | 10.0 | 0.333 | 0.193 | 0.720 |
| Internal ANABAG test set | 12.0 | 0.333 | 0.193 | 0.720 |
| External Epitope3D test set | 6.0 | 0.252 | 0.214 | 0.530 |
| External Epitope3D test set | 8.0 | 0.259 | 0.211 | 0.567 |
| External Epitope3D test set | 10.0 | 0.257 | 0.208 | 0.572 |
| External Epitope3D test set | 12.0 | 0.256 | 0.206 | 0.573 |

**Table S5**. Performance of PatchEpi on RoBep test set (144 antigens)

|  | MCC | AUPRC | F1 | Recall | Precision |
| --- | --- | --- | --- | --- | --- |
| PatchEpi on RoBep test set | 0.52 | 0.60 | 0.55 | 0.83 | 0.41 |
| RoBep on RoBep test set | 0.34 | 0.23 | 0.38 | - | - |

Table S6. Residue-level comparison of epitope labels and model predictions between 4J6R and 4YDI. Listed residues are those for which either the experimental epitope annotation or the model prediction differs between the two structures.

| Residue index | 4J6R true | 4YDI true | 4J6R pred | 4YDI pred | Comment 4J6R | Comment 4YDI |
| --- | --- | --- | --- | --- | --- | --- |
| 59_E | 0 | 1 | 1 | 1 | FP | TP |
| 82_G | 1 | 0 | 1 | 1 | TP | FP |
| 159_E | 1 | 0 | 1 | 1 | TP | FP |
| 296_R | 0 | 1 | 1 | 0 | FP | FN |
| 330_N | 1 | 0 | 1 | 1 | TP | FP |
| 342_M | 1 | 0 | 1 | 1 | TP | FP |
| 347_R | 0 | 1 | 1 | 1 | FP | TP |

# References

1.	Choi S, Kim D. B cell epitope prediction by capturing spatial clustering property of the epitopes using graph attention network. *Sci Rep* 2024;**14**(1):27496.

2.	Zeng Y, Wei Z, Yuan Q *et al.* Identifying B-cell epitopes using AlphaFold2 predicted structures and pretrained language model. *Bioinformatics* 2023;**39**(4):btad187.

3.	Steinegger M, Söding J. Clustering huge protein sequence sets in linear time. *Nat Commun* 2018;**9**(1):2542.

4.	Steinegger M, Mirdita M, Karin EL *et al.* MMseqs2 User Guide. User Guide Wiki: https://git hub. com/soedinglab/mmseqs2/wiki. url: https …, 2019.

5.	Chiu JKH, Ong RTH. Clustering biological sequences with dynamic sequence similarity threshold. *BMC Bioinformatics* 2022;**23**(1):108.

6.	Silva BM da, Myung Y, Ascher DB *et al.* epitope3D: a machine learning method for conformational B-cell epitope prediction. *Brief Bioinform* 2022;**23**(1).

7.	Dunbar J, Krawczyk K, Leem J *et al.* SAbDab: the structural antibody database. *Nucleic Acids Res* 2013;**42**(D1):D1140–6. https://doi.org/10.1093/nar/gkt1043.

8.	Capel HL, Vavourakis O, Williams BH *et al.* SAbDab2: The structural antibody database in the age of machine learning. Preprint, bioRxiv, 20 June 2026, 2026.06.16.732554. https://doi.org/10.64898/2026.06.16.732554.

9.	Cia G, Pucci F, Rooman M. Critical review of conformational B-cell epitope prediction methods. *Brief Bioinform* 2023;**24**(1):bbac567. https://doi.org/10.1093/bib/bbac567.

10.	Høie MH, Gade FS, Johansen JM *et al.* DiscoTope-3.0: improved B-cell epitope prediction using inverse folding latent representations. *Front Immunol* 2024;**15**:1322712.

11.	Brotzakis ZF, Zhang S, Murtada MH *et al.* AlphaFold prediction of structural ensembles of disordered proteins. *Nat Commun* 2025;**16**(1):1632. https://doi.org/10.1038/s41467-025-56572-9.

12.	Brandes N, Ofer D, Peleg Y *et al.* ProteinBERT: a universal deep-learning model of protein sequence and function. *Bioinformatics* 2022;**38**(8):2102–10. https://doi.org/10.1093/bioinformatics/btac020.

13.	Liu F, Yuan C, Chen H *et al.* Prediction of linear B-cell epitopes based on protein sequence features and BERT embeddings. *Sci Rep* 2024;**14**(1):2464. https://doi.org/10.1038/s41598-024-53028-w.

14.	Qi Y, Zheng P, Huang G. DeepLBCEPred: A Bi-LSTM and multi-scale CNN-based deep learning method for predicting linear B-cell epitopes. *Front Microbiol* 2023;**14**. https://doi.org/10.3389/fmicb.2023.1117027.

15.	Attique M, Alkhalifah T, Alturise F *et al.* DeepBCE: Evaluation of deep learning models for identification of immunogenic B-cell epitopes. *Comput Biol Chem* 2023;**104**:107874. https://doi.org/10.1016/j.compbiolchem.2023.107874.

16.	Chuang CC, Liu YC, Ou YY. DeepEpiIL13: Deep Learning for Rapid and Accurate Prediction of IL-13-Inducing Epitopes Using Pretrained Language Models and Multiwindow Convolutional Neural Networks. *ACS Omega* 2025;**10**(9):9675–83. https://doi.org/10.1021/acsomega.4c10960.

17.	Nguyen TT, Nguyen VN, Tran TX *et al.* Improved Linear B-Cell Epitope Prediction Using CNN and BiLSTM. In: Nghia PT, Thai VD, Thuy NT *et al.* (eds), *Advances in Information and Communication Technology*. Cham: Springer Nature Switzerland, 2025, 466–75. https://doi.org/10.1007/978-3-031-80943-9_50.

18.	Jiang F, Guo Y, Ma H *et al.* AlphaEpi: Enhancing B Cell Epitope Prediction with AlphaFold 3. *Proc 15th ACM Int Conf Bioinforma Comput Biol Health Inform* (New York, NY, USA) BCB ’24, 16 Dec. 2024:1–8. https://doi.org/10.1145/3698587.3701389.

19.	Fuchs F, Worrall D, Fischer V *et al.* Se (3)-transformers: 3d roto-translation equivariant attention networks. *Adv Neural Inf Process Syst* 2020;**33**:1970–81.

20.	Zeng S, Wang D, Jiang L *et al.* Parameter-efficient fine-tuning on large protein language models improves signal peptide prediction. *Genome Res* 2024;**34**(9):1445–54. https://doi.org/10.1101/gr.279132.124.

21.	Shuttleworth R, Andreas J, Torralba A *et al.* LoRA vs Full Fine-tuning: An Illusion of Equivalence. *ArXiv E-Prints* Oct. 2024:arXiv:2410.21228. https://doi.org/10.48550/arXiv.2410.21228.

22.	Schmirler R, Heinzinger M, Rost B. Fine-tuning protein language models boosts predictions across diverse tasks. *Nat Commun* 2024;**15**(1):7407. https://doi.org/10.1038/s41467-024-51844-2.

23.	Bax B, Blundell TL, Murray-Rust J *et al.* Structure of mouse 7S NGF: a complex of nerve growth factor with four binding proteins. *Structure* 1997;**5**(10):1275–85. https://doi.org/10.1016/S0969-2126(97)00280-3.

24.	Feng D, Kim T, Özkan E *et al.* Molecular and Structural Insight into proNGF Engagement of p75NTR and Sortilin. *J Mol Biol* 2010;**396**(4):967–84. https://doi.org/10.1016/j.jmb.2009.12.030.

25.	Leloup N, Chataigner LMP, Janssen BJC. Structural insights into SorCS2–Nerve Growth Factor complex formation. *Nat Commun* 2018;**9**(1):2979. https://doi.org/10.1038/s41467-018-05405-z.
